# Genome-wide identification of the MIKCc-type MADS-box gene family in *Chrysanthemum lavandulifolium* reveals their roles in the capitulum development

**DOI:** 10.3389/fpls.2023.1153490

**Published:** 2023-03-23

**Authors:** Junzhuo Li, Qiuling Zhang, Deyuan Kong, Ya Pu, Xiaohui Wen, Silan Dai

**Affiliations:** Beijing Key Laboratory of Ornamental Plants Germplasm Innovation and Molecular Breeding, National Engineering Research Center for Floriculture, Beijing Laboratory of Urban and Rural Ecological Environment, Key Laboratory of Genetics and Breeding in Forest Trees and Ornamental Plants of Education Ministry, School of Landscape Architecture, Beijing Forestry University, Beijing, China

**Keywords:** chrysanthemum, *Chrysanthemum lavandulifolium*, capitulum development, MIKCc-type gene family, complex inflorescences

## Abstract

*Chrysanthemum* ×*morifolium* is well known throughout the world for its diverse and exquisite flower types. However, due to the complicated genetic background of *C.* ×*morifolium*, it is difficult to understand the molecular mechanism of its flower development. And it limits the molecular breeding of improving chrysanthemum flower types. *C.* ×*morifolium* has the typical radial capitulum, and many researches showed that the members of the MIKCc-type MADS box gene family play a key role in the formation and development of the capitulum. However, it has been difficult to isolate the important MIKCc and investigate their roles in this process due to the lack of genomic information in chrysanthemum. Here, we identified MIKCc-type MADS box genes at whole genome-wide level in *C*. *lavandulifolium*, a diploid species closely related to *C.* ×*morifolium*, and investigated their roles in capitulum development by gene expression pattern analysis and protein interaction analysis. A total of 40 ClMIKCc were identified and were phylogenetically grouped into 12 clades. Members of all clades showed different enriched expression patterns during capitulum formation. We speculate that the E-class genes in *C*. *lavandulifolium* underwent subfunctionalization because they have a significantly expanded, more diverse expression patterns, and specifically tissue expression than *AtSEPs*. Meanwhile, we detected the C-class expressed in disc floret corolla, which could be the clue to explore the morphological differences between disc and ray floret corolla. In addition, the potential roles of some MIKCcs in complex inflorescence formation were explored by comparing the number and phylogenetic relationship of MIKCc subfamily members in Asteraceae with different capitulum types. Members of the FLC branch in Asteraceae were found to be possibly related to the differentiation and development of the ray floret.

## 1 Introduction

In angiosperms, flowers are the reproductive organs. The yield and quality of crop and ornamental plants are directly affected by the development of their floral organs. In *Arabidopsis thaliana*, the floral organs have been divided into four rounds according to their location and developmental sequence, which are the sepals, petals, stamens, and pistils from the outside to the inside. Each round of floral organ primordia was under the regulation of a different set of genes to determine its identity (Coen and Meyerowitz, 1991; Rounsley et al., 1995; Causier et al., 2010). Currently, the best explanation for floral organ specification in plants is the ABC(D)E model. This model assumes that the different floral primordia are synergistically regulated by several genes: sepals (A+E), petals (A+B+E), stamens (B+C+E), pistils (C+E) and ovules (D+E). The ABC(D)E model contains the A class gene *APETALLA1* (*AP1*), *APETALLA2* (*AP2*), the B class genes *PISTILLATA* (*PI*), *APETALLA3* (*AP3*), the C class gene *AGAMOUS* (*AG*), the D class genes *SEEDSTICK* (*STK*), *SHATTERPROOF1*/*2* (*SHP1*/*2*) and the E class genes *SEPALLATA1/2/3/4* (*SEP1/2/3/4*). They all belong to the MIKCc clade of the MADS box gene family except *AP2* (Becker and Theiben, 2003; Theiβen et al., 2016). Other members of the MIKCc clade have also been associated with flowering processes such as flower buds resting (Theiben et al., 2000; Wu et al., 2017; Nishiyama et al., 2021), apical meristematic differentiation (Li et al., 2010), development of inflorescence morphology (Hussin et al., 2021) and flowering time (Lee and Lee, 2010; Wang et al., 2021; Ahmar et al., 2022).

However, the members of the ABC(D)E model differ in their expression patterns in plants with more primitive floral organs or more complex inflorescence structures. In addition, their functions and regulatory mechanisms are still not well characterized (Melzer et al., 2010; Zhang et al., 2017; Zhang L. et al., 2020). The capitulum of the Asteraceae is a complex inflorescence with a highly compressed inflorescence structure. Numerous florets are borne in a spiral pattern on the capitulum in a Fibonacci series with the involucre wrapped around the outside. They can be classified as homogeneous capitulum (discoid or ligulate type) and heterogeneous capitulum (radiate type) depending on the type of florets borne on the receptacle. The capitulum is the most peculiar and important organ of Asteraceae plants, gives them a unique ornamental value. Members of the MIKCc subfamily have been shown to be essential for capitulum development in numerous studies (Elomaa et al., 2018).

Although the capitulum of Asteraceae is known as pseudanthium, the process of developing floral organs and the function of ABC(D)E-class genes in the capitulum differs from that of the single flower (Elomaa et al., 2018; Zhang and Elomaa, 2021). The involucre replaces the first floral organ (sepals) to protect the inner floral organs. This results in the disappearance of the sepals or their transition to crown hairs in the capitulum to aid seed dispersal. The A-class gene *AP1* regulates the initiation and development of crown hairs in *Taraxacum mongolicum*. However, it is also widely expressed throughout the capitulum (Bremer, 1994; Harris, 1995; Wen et al., 2019a; Vijverberg et al., 2021). The structure and morphology of the second floral organ (petal) differ between the two types of florets in the radiate capitulum: Peripheral ray florets mimic the petals of individual flowers to attract pollinators. These petals are ligulate with complete upper and lower epidermis and parenchymatous tissue. The disc florets are responsible for receiving pollen and reproducing, with their petals enclosed in a corolla tube without parenchymatous tissue. B-class genes that determine petal identity are differentially expressed in the corollas of two floret types in *C*. *lavandulifolium* and *C*. ×*morifolium*, with *AP3*/*PI* both highly expressed in the ray floret corollas (Wen et al., 2019a). *GDEF2* and *GDEF3*, the orthologous genes of *AP3* in *Gerbera hybrida*, have a broader domain of expression during the differentiation of the floral organs (Broholm et al., 2010). The third floral organ (stamen) also differs in the two types of florets in the radial capitulum. Disc florets are bisexual and ray florets have only one pistil. The C class gene *AG*, which determines stamen identity, is conserved in two copies in *G*. *hybrida*, *C. lavandulifolium*, and *C.* ×*morifolium* with radial capitulum. However, the expression patterns in these three species are different (Yu et al., 1999; Wen et al., 2019a). All florets on the capitulum have a normally developed fourth floral organ (pistil) and ovule. In *Tagetes erecta*, *TeAGL11-1* shows a partial function of the D-class genes that regulate seed and petal development (Zhang C. et al., 2020). The D-class gene showed more novel function except control the seed development in Asteraceae species. A total of seven *SEP*-like genes were identified in *G*. *hybrida*, indicating a significant expansion of the SEP evolutionary branch. SEPs are highly functionally redundant in *A. thaliana*, but in *G*. *hybrida* they showed specific transcriptional patterns during capitulum formation and floral organ identity determination, suggesting subfunctionalization and neofunctionalization (Zhang et al., 2017). Thus, the relatively conserved expression patterns and functions of ABC(D)E class genes in capitulum and simple plants have resulted in the pseudanthium formation. Meanwhile, their general expanded, differentiated and subfunctionalized in Asteraceae have led to significant differences in the floral organs of capitulum and simple plants. In the meantime, ABC(D)E class genes often regulate floral organ differentiation and development as protein tetramers. Their protein interactions in Asteraceae need to be further investigated.

MIKCc members showed significant neofunctionalization during capitulum and floret development in Asteraceae compared to model plants. *ClCAL* and *ClFUL* are specifically highly expressed in *C. lavandulifolium* at the stage of floret primordia initiation. They may be involved in the differentiation of ray and disc florets (Wen et al., 2022). The B class gene *CmPI*/*CmAP3* is differentially expressed in the ray and disc florets of *C.* ×*morifolium*. It leads to the localization of carotenoid accumulation in the petals (Lu et al., 2022). The C-class gene *ScAG* and the D-class gene *ScAGL11* can inhibit anthocyanin synthesis in ray florets of *Senecio cruentus*, which in turn affects the formation of flower spots (Qi et al., 2022). *AGL6* plays the role of the A class gene in basal angiosperms (Kim et al., 2005). However, *GhGRCD3*, the *AGL6* ortholog in *G*. *hybrida*, functions as both an A- and E-class gene, affecting crown hair and corolla identity and development, as well as inflorescence meristem maintenance and floral meristem differentiation (Ruokolainen et al., 2010a; Zhang et al., 2017). The key lowering integrator *SUPPRESSOR OF OVEREXPRESSION OF CO1* (*SOC1*) affects ray floret development in *G*. *hybrida*. Overexpression of *GhSOC1* causes ray floret shortening and discoloration (Ruokolainen et al., 2011). The current research indicates that MIKCc-type MADS box genes have a relatively conserved function during flower development in angiosperms. However, due to the complex structure of the capitulum, ABC(D)E-like genes appear to be commonly expanded, neofunctionalized, and subfunctionalized in Asteraceae. Other MIKCc genes have unique functions in capitulum formation and development. Therefore, the potential new functions of these classical flower development genes and their roles in the process of capitulum formation can be discovered through the mining, identification, and functional studies of MIKCc in Asteraceae.

Recently, with the release of the Asteraceae genome information (Badouin et al., 2017; Reyes-Chin-Wo et al., 2017; Song et al., 2018; Liu et al., 2020; Nakano et al., 2021; van Lieshout et al., 2022; Wen et al., 2022), the identification of gene families based on a genome-wide level has become feasible. Here, *C*. *lavandulifolium* (2n=2*x*=18) was selected as the object of study. It’s a perennial herb and a closely related diploid of the cultivated chrysanthemum (*C*. ×*morifolium*), often used as a typical material in chrysanthemum studies. In our previous work, we have conducted a detailed study of the capitulum development process of *C. lavandulifolium*, and the study of the MIKCc gene family of *C. lavandulifolium* will help us to further elucidate its formation mechanism (Wen et al., 2019a; Wen et al., 2019b; Wen et al., 2022). In this study, the MIKCc-type MADS box gene family members in the *C. lavandulifolium* genome been identified. We analyzed their protein physicochemical properties, conserved motifs, and phylogenetic relationships. Furthermore, based on transcriptome data, we screened for highly expressed ClMIKCcs during *C*. *lavandulifolium* capitulum and floral organ development. And qRT-PCR, yeast two-hybrid (Y2H) and luciferase complementation assay (LCA) were used to analyze the expression patterns and protein interactions of these ClMIKCcs in nine floral organs of *C. lavandulifolium*. These results will help to gain insight into the specific functions of the MIKCc-type MADS-box gene in complex inflorescences, and the pattern of the ClMIKCc protein complex. They will also provide potential genetic resources for floral shape improvement in *C*. ×*morifolium*. Based on this, we further compared the contraction/expansion of MIKCc subfamily members in Asteraceae, discussed the differentiation characteristics of these members in different capitulum type Asteraceae plants, and provided some new perspectives for resolving MIKCc evolution correlated with capitulum types in Asteraceae.

## 2 Materials and methods

### 2.1 Collection of publicly datasets and identification of MIKCc-type MADS-box genes

A total of 16 species, including *A. thaliana* as a reference species, and 15 Asteraceae with three different capitulum types were examined in our study to determine whether the number and classification of MIKCc genes correlated with capitulum type. Among these Asteraceae, radiate type capitulum with both ray florets and disc florets such as *Artemisia annua*, *C. lavandulifolium*, *C. makinoi*, *C. nankingense*, *C. seticuspe*, and *Helianthus annuus*, *Stevia rebaudiana*, and *Smallanthus sonchifolius*. Capitulum of the discoid type with disc florets only, such as *Arctium lappa*, *Cynara cardunculus*, *Carthamus tinctorius*, and *Mikania micrantha*. Capitulum of the ligulate type with only ray florets such as *Cichorium endivia*, *Taraxacum kok*-*saghyz*, and *Lactuca sativa*.

The reference sequences of *A. thaliana* MIKCc-type transcription factors were available from the Plant Transcription Factor Database (http://planttfdb.gao-lab; Tian et al., 2020). The genome data of *A. annua* (PRJNA280557), *Arctium lappa* (PRJNA764011), *C. cardunculus* (PRJNA453787), *C. lavandulifolium* (PRJNA681093), *C. endivia* (PRJNA797903), *C. tinctorius* (PRJNA313459), *M. micrantha* (PRJNA528368), *S. rebaudiana* (PRJNA436363), and *S. sonchifolius* (PRJNA798108) were downloaded from the NCBI (https://www.ncbi.nlm.nih.gov/). The genome data of *H. annuu* (PRJNA345532), *L. sativa* (PRJNA173551), were downloaded from the EnsemblPlants (http://plants.ensembl.org/index.html). The genome data of *T._kok-saghyz* (PRJCA005187) was downloaded from Genome Warehouse (https://ngdc.cncb.ac.cn/gwh/). The genome data of *C. nankingense* was downloaded from Chrysanthemum Genome Database http://www.amwayabrc.com/zh-cn/index.html). *C. makinoi* genome data was downloaded at https://www.chrysanthemumgenome.wur.nl/. And *C. seticuspe* (Gojo-0 v1) genome data was downloaded from the Plant Garden database (https://plantgarden.jp/en/list/t1111766/genome/t1111766.G002).

AtMIKCc was used as a reference sequence to search against the genomic protein sequences using TBtools v1.098765 with an E-value cut-off of 1e^-5^ (Chen et al., 2020) to confirm the candidate MIKCc-type MADS-box genes in the above species. Each predicted sequence was then checked against NCBI and the redundant sequences were removed.

The transcriptome data of *C. lavandulifolium* (SRR14723013-SRR14723033) were downloaded from NCBI (https://www.ncbi.nlm.nih.gov/). The six stages represent the six key stages of *C. lavandulifolium* capitulum development: During the vegetative stage, there are no primordia on the conical-shaped shoot apical meristem (SAM) (stage 1, S1). During the short-day condition, SAMs began to change from conical shape to hemispherical shape (stage 2, S2). At the early stage of floret primordia, only the first-round disc floret primordia differentiated at the margin, which is a key stage for the differentiation of disc and ray florets (stage 5, S5). At the middle stage of floret primordia differentiation, ray floret primordia initiated between the involucre and the outermost disc floret primordia (stage 6, S6). At the middle stage of corolla primordia differentiation, ray floret corollas began to initiate, while disc florets differentiated to produce stamen primordia and pistil primordia (stage 9, S9). At the final stage of corolla primordia differentiation, the corolla formation of the disc florets and the ray florets was completed, and they maintained a closed state (Stage10, S10) (Wen et al., 2019b).

### 2.2 Chromosomal location, phylogenetic analysis, conserved structural domains and conserved motifs analysis

The chromosomal location of ClMIKCc was obtained from genome annotation files (Wen et al., 2022), and TBtools v1.098765 (Chen et al., 2020) was used to map their physical location on *C. lavandulifolium* chromosomes and display the co-linearity.

The maximum likelihood (ML) method and 5000 bootstrap replicates were used to construct the phylogenetic tree of MIKCc. TBtools v1.098765 (Chen et al., 2020) was used. In this part, sequence alignments were performed with MUSCLE (Edgar, 2004), and trimAI (–automated1 option, Capella-Gutiérrez et al., 2009) helped us trim poorly aligned sequences and reserve reliable comparison results. The best-fit model (JTT+R7) chosen according to BIC and used to construct the phylogenetic tree in IQtree v2 (Minh et al., 2020). The results were further graphically edited using iTOL (https://itol.embl.de/).

The Conserved Domains Search database (https://www.ncbi.nlm.nih.gov/cdd/?term=) was used to predict the conserved domains of MIKCc. The predicted conserved domains were visualized using TBtools v1.098765 (Chen et al., 2020).

The MEME (https://meme-suite.org/meme/tools/meme) was used to identify the conserved motifs in AtMIKCc and ClMIKCc with the following optimal parameters: the width of the motifs was from 6 to 50 amino acids and the maximum number of motifs was 10. TBtools v1.098765 (Chen et al., 2020) was used to visualize the identified motifs.

### 2.3 Physicochemical properties analysis and subcellular localization prediction

The molecular weight and theoretical pI of AtMIKCc and ClMIKCc were analyzed using the Expasy ProtParam online software (https://web.expasy.org/protparam/), and the subcellular localization was predicted using the WOLF PSORT online tool (https://wolfpsort.hgc.jp/).

### 2.4 RNA extraction and gene expression measurement

**Figure 1:**
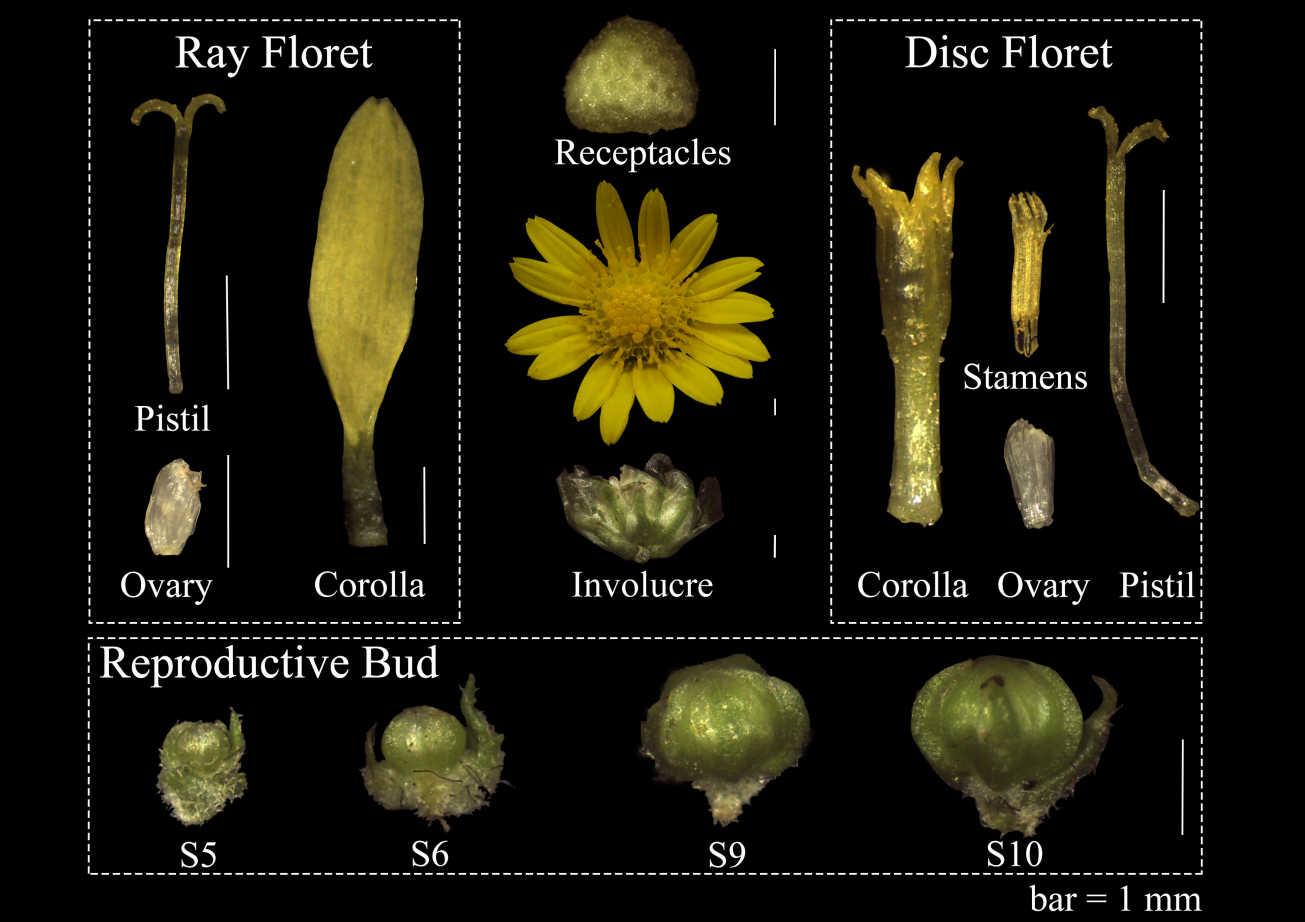
Floral organs on capitulum and four development stages reproductive bud in *C. lavandulifolium* for qRT-PCR. bar=1mm.

The genome-sequenced *C. lavandulifolium* G1 line was cultured at Beijing Forestry University for gene expression analysis (Wen et al., 2022). These materials were planted in 10 × 9 cm plastic pots with peat: vermiculite = 1:1. The temperature was 22 ± 1 °C and the light intensity was 4000 lx. The nutritive growth was carried out under long daylight (16 h light/8 h dark). After they grew 14 leaves, they were transferred to short daylight (12 h light/12 h dark) for reproductive growth (Fu et al., 2014). All floral organs (involucre, receptacles, corolla, pistils, ovules, stamens) were sampled at flowering, and four developmental stages of reproductive buds were collected based on Wen’s literature (Wen et al., 2019b). The morphology of the sampled material is shown in **Figure 1**. All the material was immediately frozen in liquid nitrogen and stored at -80°C for the extraction of total RNA.

Total RNA was extracted from the collected plant materials using Plant RNA Rapid Extraction Kit (HUAYUEYANG Biotechnology, Beijing, China) and treated with RNase-free DNaseI to digest DNA. Gene expression analysis was performed by Real-time quantitative reverse transcription-PCR (qRT-PCR), which was performed using a Mini Opticon Real-time PCR System (Bio-Rad Laboratories Inc., Hercules, CA, USA) based on the SYBR Premix Ex Taq (Takara Bio Inc., Shiga, Japan). Three biological replicates were used to confirm the reliability of the results. *ClSAND* was used as an internal control gene for qRT-PCR (Qi et al., 2016). The primers for qRT-PCR were shown in **Supplementary Table 2**. The ΔΔCt method was used to analyze qRT-PCR data.

### 2.5 Protein-protein interaction assay

Pairwise yeast two-hybrid analyses were performed to verify the interactions between ClMIKCc. Gene-specific primers (**Supplementary Table 3**) were used to amplify protein-coding regions for all ClMIKCc tested. The PCR products were then fused into pGADT7 and pGBKT7 vectors. Sequencing was performed to verify the fusion. The constructs were then transformed into the Saccharomyces cerevisiae strain Y2H Gold and sequenced. Only the ClPI and ClSEPd constructs were found to have the ability to self-activate the expression of the HIS3 reporter genes. 10 mmol/L 3-amino-1, 2, 4-triazole (3-AT) was used to eliminate self-activation. For further interaction studies, recombinant vectors were used. Three technological replicates and three different concentrations were used in the protein-protein interaction experiments.

### 2.6 Luciferase complementation assay

The luciferase complementation assay (LCA) was used to verify the positive results obtained in the Y2H assay (Chen et al., 2008). Target genes were ligated separately to pCAMBIA1300-cLUC and pCAMBIA1300-nLUC using the homologous recombination method. All primer sequence used to construct the vector is shown in **Supplementary Table 4**. Stop codon removal was required for all genes ligated to pCAMBIA1300-nLUC. The resulting recombinant plasmid was transformed into *Agrobacterium tumefaciens* (GV3101) using the freeze-thaw method.

Empty pCAMBIA1300-cLUC and pCAMBIA1300-nLUC carriers were used as controls. Subsequently, the Agrobacterium suspension (OD600 = 1.0) was injected into the abaxial leaf surface of three-week-old *Nicotiana benthamiana* leaves. After 72 h (24 h dark and 48 h light)of incubation in the greenhouse, D-Luciferin potassium salt was applied to the abaxial side of these leaves and then placed in the dark for 5 min. The fluorescence signal was observed using LB983 NightOwl II (Berthold Technologies, Bad Wildbad, Germany).

## 3 Results

### 3.1 Identification and general information of ClMIKCc

The 42 possible MIKCc proteins were retrieved from the *C. lavandulifolium* genome using two different BLAST methods. The retrieved MIKCc proteins were further screened based on the results of multiple sequence comparisons and the Conserved Domain of the NCBI website. The final 40 ClMIKCc proteins were identified. ClMIKCc encodes a polypeptide of 183 to 296 amino acids ranging in molecular weight from 20.73 to 34 kD. The theoretical pI range was 5.07 to 9.58. Subcellular localization predictions showed that 32 of the 40 ClMIKCc proteins were localized in the nucleus, which is typical of transcription factors (**Supplementary Table 1**).

**Figure 2:**
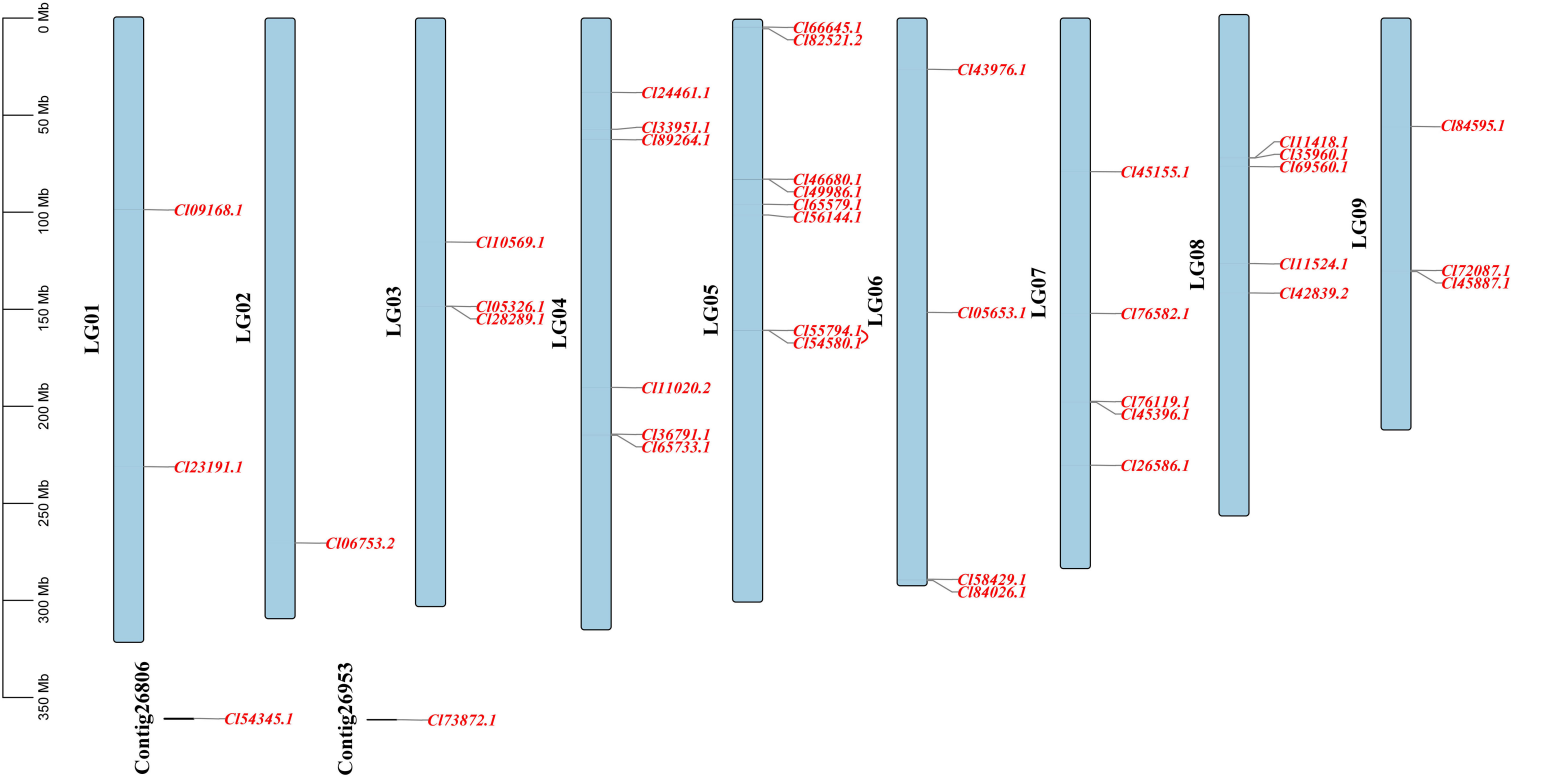
Localization and co-linearity of *ClMIKCc* on nine *C. lavandulifolium* chromosomes. The red line shown the co-linearity relationship between genes.

Based on the genome annotation information of *C. lavandulifolium*, 38 of the 40 ClMIKCc were localized to all nine chromosomes. Two ClMIKCc were not associated with chromosomes (**Figure 2**). These ClMIKCc were not evenly distributed among the chromosomes. Chromosomes 1, 2, and 9 contained the fewest members, with two members each, and chromosome 5 contained the most, with eight members. The co-linearity analysis shows that only two MIKCc undergone a tandem repeat (**Figure 2**).

### 3.2 Phylogenetic analysis of ClMIKCc and AtMIKCc

**Figure 3:**
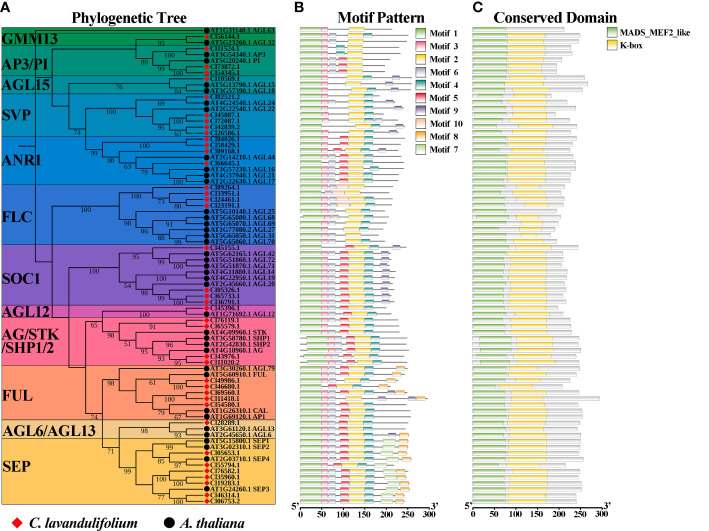
Analysis of phylogenetic, conserved motifs, and conserved structural domains of ClMIKCc and AtMIKCc. **(A)** ML phylogenetic tree of ClMIKCc and AtMIKCc. The number above branches represented bootstrap value and the values below 50 would not be shown; **(B)** Conserved motifs in ClMIKCc and AtMIKCc, 10 predicted motifs are indicated by different colored squares; **(C)** Conserved structural domains in ClMIKCc and AtMIKCc, 2 predicted structural domains are indicated by squares of different colors.

To elucidate the phylogenetic relationships of ClMIKCc, an ML phylogenetic tree was constructed using 40 ClMIKCc and 39 AtMIKCc (**Figure 3A**). ClMIKCc were distributed in 12 clades (GMM13, AP3/PI, AGL15, SVP, ANR1, FLC, SOC1, AGL12, AG/SHP/STK, AP1/FUL, AGL6, SEP), ClMIKCc underwent contraction in FLC and SOC1 clade and expansion in SVP, FUL and SEP clade.

Using the MEME website, the conserved motifs of ClMIKCc and AtMIKCc were predicted. 10 conserved motifs were identified and labeled as motifs 1-10 (**Supplementary Figure 1**). For ClMIKCc and AtMIKCc, the motifs were relatively consistent and were located in the same clade (**Figure 3B**). There are 4-8 conserved motifs in each ClMIKCc, and all ClMIKCc and AtMIKCc proteins have Motif 1 and Motif 2. Combined with the predicted results of conserved structural domains, Motif 1 and Motif 2 are the core motifs of the MADS domain and K-box, respectively. Motif 7 is a specific conserved motif in At- and ClSEPs. Motif 10 is present only in ClFLCs (**Figure 3C**, **Supplementary Figure 1**).

### 3.3 Expression pattern of ClMIKCc during *C. lavandulifolium* capitulum development

**Figure 4:**
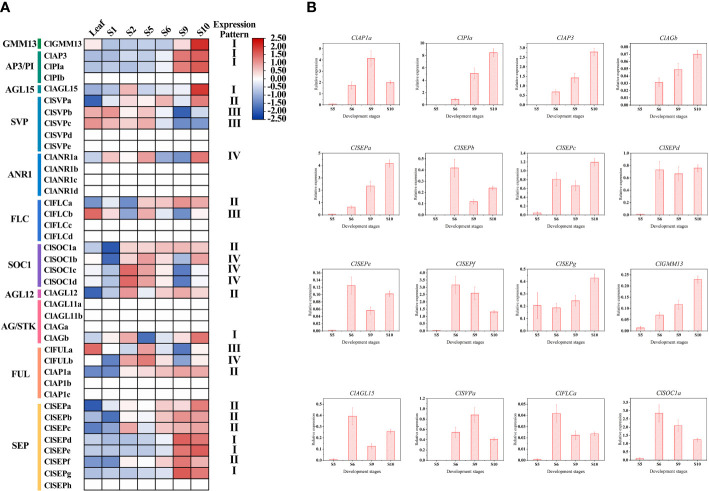
Expression pattern of *ClMIKCc* during the capitulum development stage. **(A)** Expression heat map constructed based on transcriptome data during the capitulum development stage of *C. lavandulifolium*. **(B)** qRT-PCR analysis of the expression level of class I and class II ClMIKCc in four development stages. Error bars represent the standard errors of three biological replicates.

Based on the public transcriptome dataset of *C. lavandulifolium* capitulum developmental stages (Wen et al., 2022), the expression pattern of *ClMIKCc* during *C. lavandulifolium* capitulum development was analyzed. A total of 26 *ClMIKCc* are expressed in leaves and six developmental stages (**Figure 4A**). These *ClMIKCc* could be mainly classified into four classes according to their expression patterns. Among them, class I, class II, and class IV *ClMIKCc* were specifically expressed in the capitulum (**Supplementary Figure 2**). Class I included the B-, C-class genes and *ClSEPd/e/f*, which were abundantly expressed in the late stage of capitulum development (floral organ differentiation period). These genes are mainly involved in the process of floral organ differentiation in *C. lavandulifolium* florets. Class II includes *ClAP1a*, *ClSEPa/b/c/f*, *ClSOC1a*, *ClSVPa* and *ClFLCa*, which showed high expression levels after the floral transition. They may be involved in both inflorescence primordium development and floral organ differentiation. Class III includes *ClFLCb*, *ClFULa*, and *ClSVPb/c*, which are mainly expressed in the leaf, the apical meristem before and after the floral transition, and the early stage of capitulum development. They may be involved in the floral transition process in *C. lavandulifolium*. Class IV contains *ClANR1a*, *ClSOC1b/c/d* and *ClFULb*, which are mainly expressed in the middle stage of capitulum development (S2-S6). They may regulate the floral transition and the formation of the inflorescence structure.

To further elucidate the role of *ClMIKCc* in the development of the *C. lavandulifolium* capitulum, we examined the expression of class I and class II *ClMIKCc* in the capitulum of *C. lavandulifolium* at four critical periods after the initiation of the florets primordium by qRT-PCR. As shown in **Figure 4B**, almost all of these *ClMIKCc* were first expressed after the initiation of the floret primordium (S5). The expression increased with increasing floret number and floral organ differentiation. Interestingly, with the flowering process of *C. lavandulifolium*, the expression of *ClSVPa* and *ClFLCa*, two genes usually considered as flowering repressors, were also up-regulated. During the development of the *C. lavandulifolium* inflorescence, they may play a different role than their paralogous genes.

### 3.4 ClMIKCc expression and protein interaction in *C. lavandulifolium* floral organs

**Figure 5:**
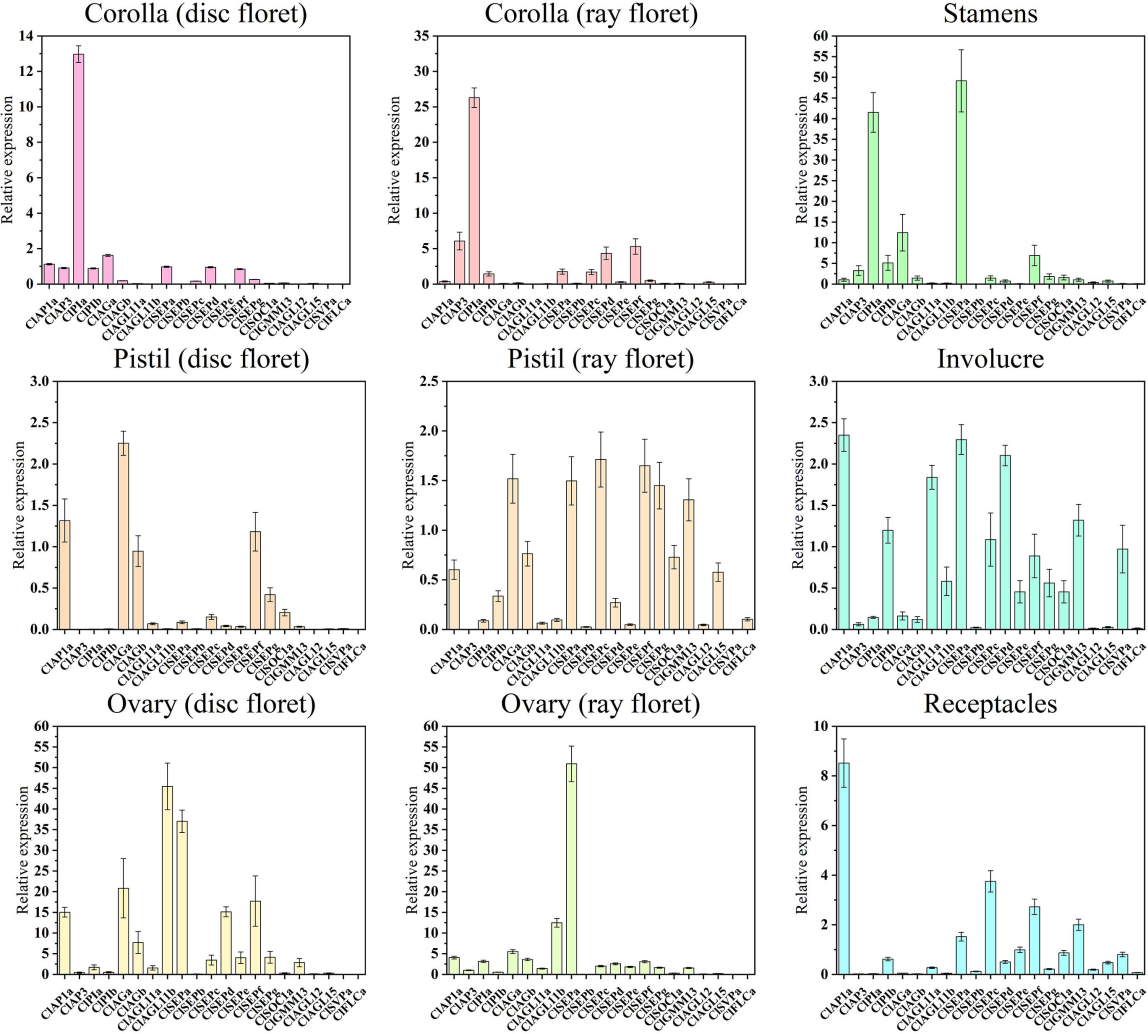
Quantitative RT-PCR analysis of the expression level of 21 ClMIKCc in nine floral organs. Error bars represent the standard errors of three biological replicates.

In order to investigate these genes’ roles in the development of different floral organs, 21 *ClMIKCc* was determined by qRT-PCR in nine floral organs. Class I and II of *ClMIKCc* were accompanied by the floral organ development, highly expression during the S9 and S10. Those ClMIKCc may be involved in the process of floral organ identity determination and development. The results (**Figure 5**) showed that the A-class gene *ClAP1a* was widely expressed in the nine floral organs. There are three B-class genes in *C. lavandulifolium*, *ClAP3*, *ClPIa*, and *ClPIb*, among which *ClAP3* and *ClPIa* had a typical B-class gene expression pattern that expressed mainly in the stamens and corollas, also a weak expression was detected in the ovules. However, *ClPIb* was detected weakly expressed in all floral organs. A total of two C-class genes were identified in the *C. lavandulifolium* genome, *ClAGa* was not only expressed in the pistil, ovule, and stamen but also partially overlapping expressed with *ClAP1a* in the corolla of disc floret. *ClAGb* showed a low expression level and a broad expression domain which is like *ClPIb*. This expression patterns difference of paralogous genes was also present in two *ClAGL11*-like genes, *ClAGL11a* specifically expressed in the ovules, while *ClAGL11b* expressed in all floral organs but with a lower level. The E-class genes in the SEP branch showed diverse expression patterns. *ClSEPa* was mainly expressed in ovules and stamens. *ClSEPc* expressed in all floral organs except corollas and pistils of disc floret. *ClSEPd* and *ClSEPe* mainly expressed in ovules and ray floret corollas. *ClSEPb*, *ClSEPf*, and *ClSEPg* expressed in all floral organs.The result also showed that *ClAGL12*, *ClAGL15*, and *ClFLCa* weakly expressed in all floral organs. *ClGMM13* and *ClSOC1a* expressed in pistils, carpels, stamens, involucre, and receptacles. C*lSVPa* specifically expressed in involucre and receptacles (**Figure 5**).

The expression pattern analysis of 26 *ClMIKCc* in nine floral organs showed that they had similar patterns in the corresponding floral organs between the two floret types (**Figure 5**). The expression patterns of *ClMIKCc* in the floral organs of *C. lavandulifolium* were generally consistent with the ABC(D)E model in model plants (**Figure 5**). The B-and E-class genes were predominant in the corolla of both floret types. The stamen development was regulated by B-, C-, and E-class genes. And the pistils and ovules development were regulated by B-, C-, and E-class genes as well as *AGL11*-like. However, differences in the expression level of same genes or the tissue-specific expression patterns of homologous genes in two floret types may be the cause of their morphology distinction.

**Figure 6:**
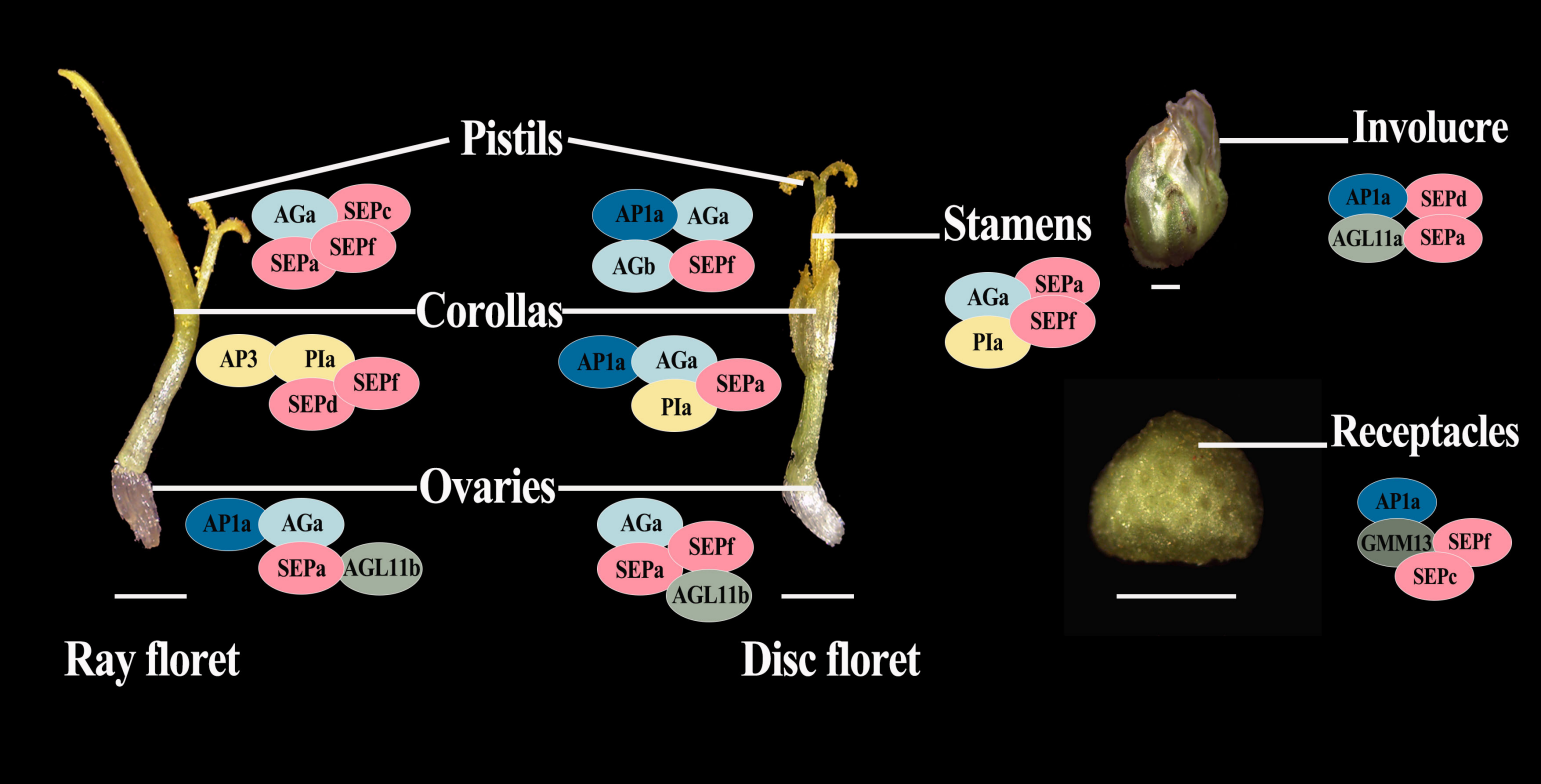
Suppositional interaction model of the four most highly expressed ClMIKCc in the nine floral organs of *C. lavandulifolium*. Different colors are used to distinguish the members of the different clades. Intersecting ellipses indicate that the protein pair has interactions. All positive results are supported by the Y2H and LCA results. bar=1 mm.

Since members of the MIKCc gene subfamily usually form homologous or heterologous protein complexes to function, we performed the yeast two-hybrid assay and LCA to analyze the protein interaction between the four most highly expressed ClMIKCc in each floral organ. The positive results obtained for all Y2H systems are shown in the **Supplementary Figure 3**. Since the Y2H system may give false positive results, we verified the positive results of the heterologous interactions by the LCA in tobacco (**Supplementary Figure 4**). These results showed that these four ClMIKCc were able to form protein complexes through different modes of interaction (**Supplementary Figure 3**-**4**). Although the A-class gene *ClAP1a* was widely expressed in the capitulum, it could only interact with ClAGa, ClAGb, and ClAGL11b from the AG clades, as well as the homolog of Bsister (Bs), ClGMM13 (**Supplementary Figure 3**). Among the B class genes, ClAP3 can only interact with ClPIa. However, ClPIa can extensively form protein dimers with C and E class genes. Similar to B class genes, among two copies of C class gene ClAG, ClAGa was able to form protein dimers with more ClMIKCc. ClAGb was only able to interact with A and C class genes. ClAGL11-like is highly expressed in ovules and can interact with E-class genes. Therefore, it may function as a D-class gene in *C. lavandulifolium*. *ClSEPs* are highly expressed in all floral organs, and they also interact to a large extent with members of other clades. Thus, they appear to be key members of the multi-protein complex. ClGMM13 from the Bs evolutionary branch can interact with A- and E-class genes. Its function may also depend on the formation of the protein complex (**Figure 6**).

### 3.5 Evolutionary analysis of MIKCc in Asteraceae with different capitulum types and single flower plants

**Figure 7:**
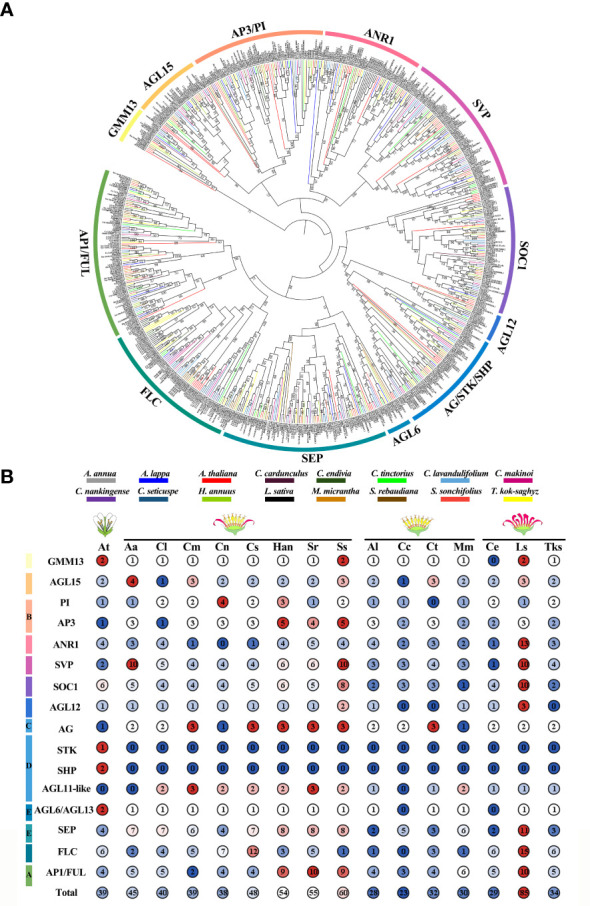
Phylogenetic analysis and statistic of MIKCc in Arabidopsis and fifteen Asteraceae. **(A)** The ML Phylogenetic tree of MIKCc in 15 Asteraceae. The bootstrap replicates were 5000. The number above branches represented bootstrap value and the values below 50 would not be shown. We used different colors to distinguish species and clades. **(B)** The member counts of each clade of MIKCc in 16 species. Their respective inflorescence types are indicated on the top. At, *A. thaliana*; Aa, *A. annua*; Cl, *C. lavandulifolium*; Cm, *C. makinoi*; Cn, *C. nankingense*; Cs, *C. seticuspe*; Han, *H. annuus*; Sr, *S. rebaudiana*; Ss, *S. sonchifolius*; Al, *A. lappa*; Cc, *C. cardunculus*; Ct, *C. tinctorius*; Mm, *M. micrantha*; Ce, *C. endivia*; Ls, *L. sativa*; Tks, *T._kok-saghyz*.

For Asteraceae MIKCc phylogenetic analysis, ML trees were constructed using MIKCc proteins from 16 species. These MIKCcs were grouped into 12 clades, and the number of proteins varied greatly among these branches. The SVP, SOC1, SEP, FLC, and AP1/FUL clades had more members (**Figure 7A**). Furthermore, the number of MIKCc per species in each branch was counted (**Figure 7B**). The results showed that the number of MIKCc was lowest in *C. cardunculus*, which has a discoid type capitulum, with 23 members. While the highest number of proteins was found in *L. sativa*, which had a ligulate type capitulum, with 85 members. SEP clade significant expansion in radial capitulum species, the number generally more than discoid and ligulate type capitulum species. This may be related to the more complex inflorescence structure of the radial capitulum. FLC clade is abundantly retained in Asteraceae with ray floret, whether they were need vernalization.

## 4 Discussion

### 4.1 Overview of MIKCc-type MADS-box gene family in *C. lavandulifolium*

A total of 40 MIKCc-type MADS box genes were identified from the whole genome sequence of *C. lavandulifolium*. Chromosome localization results showed that ClMIKCcs were unevenly distributed on nine chromosomes of *C. lavandulifolium* (**Figure 2**). Based on a phylogenetic tree constructed from ClMIKCc and AtMIKCc, the ClMIKCcs were divided into 11 clades (**Figure 3A**). The number of A-class genes *AP1*, B-class genes *PI\AP3*, C-class genes *AG*, and flower-forming integrator *SOC1* genes in *C. lavandulifolium* genome was consistent with previous results based on the *C. lavandulifolium* transcriptome and homologous clones (Wang et al., 2014; Wen et al., 2019a). In addition, we identified many E-class genes in the *C. lavandulifolium* genome. These SEP clade members showed diverse expression patterns and extensive protein interactions during capitulum and floral organ development (**Figures 4**–**6**), which may play a critical role in regulating complex inflorescence formation.

Although *C. lavandulifolium* does not require vernalization to complete floral transition (Fu et al., 2014), the flowering repressor *FLOWERING LOCUS C* (*FLC*), the key integrator of the vernalization pathway, retains four members in the *C. lavandulifolium* genome. In contrast, other nonvernalizing plants such as *Oryza sativa*, *Cucumis sativus*, *Zea mays*, *Sorghum bicolor*, and *Gossypium hirsutum* have experienced a complete loss of members of the FLC members (Arora et al., 2007; Zhao et al., 2011; Hu and Liu, 2012; Ren et al., 2017). Notably, all ClFLCs have a unique motif 10 (**Figure 3B**, **Supplementary Figure 1**). This motif is not found in other ClMIKCcs and all AtMIKCcs. These results suggest that the ClFLCs may have a specific function in the flowering process of *C. lavandulifolium*.

### 4.2 Role of ClMIKCc in the *C. lavandulifolium* capitulum development

In *A. thaliana*, members of the MIKCc subfamily not only regulate floral organ identity and development, but also play important roles in several biological processes, such as flowering time, nitrogen utilization, and seed development (Gan et al., 2005; Mizzotti et al., 2014; Theiβen et al., 2016). ClMIKCc has a typical MADS box domain and K box, with similar conserved motifs to the *A. thaliana* reference sequence (**Figure 3**), and its function may also be conserved.

However, unlike *A. thaliana*, the process of capitulum development in *C. lavandulifolium* involves five stages, which are floral induction, floral transition, involucre differentiation, floral primordia differentiation, and corolla primordia differentiation (Wen et al., 2019b). We found that *ClAP1*, *ClAP3*, *ClPIa*/*b*, *ClAGb*, *ClSEPs*, *ClSOC1a*, *SHORT VEGETATIVE PHASE* (*ClSVPa*), and *ClFLCa* maintained a high level of expression after the floral primordia (**Figure 4B**). In *A. thaliana*, *AP1* is an important floral meristem maintenance gene to maintain the limited growth of floral meristem tissues (Pelaz et al., 2001; Litt, 2007; Han et al., 2014), in addition to functioning as an A-class gene to determine the identity of sepals and petals. In *G. hybrid*, the A- and E-class genes work together to determine the fate of the inflorescence meristem tissue (Uimari et al., 2004; Ruokolainen et al., 2010b). Usually, *SOC1* considered an activator in the flowering pathway, and *SVP* and *FLC* were repressors (Blümel et al., 2015). In *A. thaliana*, *AGL24*, *SVP* and *AP1* function together to promote and maintain the floral meristem (Gregis et al., 2008). *SOC1*. In contrast, *FLC* were not found to be associated with inflorescence meristem or floral meristem formation. In view of the fact that there are specific motifs in *ClFLCs* (**Figure 2B**) and *ClFLCa* expression of which is upregulated with *C. lavandulifolium* flowering (**Figure 4B**), we hypothesize that part of *FLCs* are involved in the formation of the inflorescence meristem and floral meristem.

In this study, we found some important genes regulating the developmental process of *C. lavandulifolium* capitulum, including classical A-, B-, C- and E-class genes. Novel expression patterns were also found for some key genes in the photoperiodic pathway. However, their function needs to be further investigated.

### 4.3 Expression pattern and interaction model of ClMIKCc in *C. lavandulifolium* floral organs

The involucre is formed by a cluster of bracts. It is an important basis for the classification of Asteraceae (Carlquist, 1976), based on its shape and the number of whorls. The involucre is located on the outermost whorl of the capitulum. It envelops and protects the capitulum (Yang and Sun, 2009). It is similar to the first whorl of the floral organs (sepals) in individual flowers in morphology, position, and function. We observed a high expression level of A- and E-class genes expressed in the involucre of *C. lavandulifolium* (**Figure 5**). This expression pattern is also similar to that of sepals (Theißen et al., 2016). Interestingly, we also found that the D-class gene *ClAGL11a* is highly expressed in the involucre (**Figure 5**). It can also interact with ClAP1a and ClSEPa to form a protein complex (**Figures 5**, **6**). In contrast, ectopic expression of STK/AGL11 in *A. thaliana* leads to the transformation of sepals into carpel-like organs with ovules, indicating the antagonism between D-class genes and sepals (Favaro et al., 2003; Pinyopich et al., 2003). This result suggests that the involucre of *C. lavandulifolium* is similar to the first whorl of floral organs (sepals) of *A. thaliana*, but not in terms of the pattern of gene expression. The A-class gene *ClAP1a* is widely expressed in all floral organs. This result is similar to previous findings in *C. lavandulifolium*, *C*. ×*morifolium*, and *G*. *hybrida* (Ruokolainen et al., 2010b; Wen et al., 2019a).

There is a clear difference in corolla morphology between ray and disc florets (**Figure 1**). B class genes have a higher expression level in the ray floret corolla. Interestingly, we observed an overlap in the expression of A- and C-class genes in the disc floret corolla of *C*. *lavandulifolium* (**Figure 5**). C-class genes are usually considered to have a negative effect on corolla formation (Wollmann et al., 2010). In the classical ABC model, the A- and C-class genes are antagonistic to each other, with the A-class genes repressing the expression of the C-class genes in order to maintain petal/stamen differentiation (Coen and Meyerowitz, 1991). However, in the disc floret corolla, *ClAP1a* and *ClAGa* not only have similar expression patterns. They can also form a heterodimer. We suggest that *ClAGa* may be a candidate to explain such differences, considering the obvious differences in length and structure between ray and disc corollas. Meanwhile, E-class genes are also differentially expressed between the two floral types (**Figure 5**). Studies on *G. hybrid* have also shown that E-class genes can directly affect floral organ identity and morphology (Zhang et al., 2017).

Except for the C class genes that were found to be expressed in the corolla of disc florets, the B, C, D, and E class genes in *C*. *lavandulifolium* have similar tissue-specific expression to the model plant (**Figure 5**). We also found that the retention pattern of *C*. *lavandulifolium* is conserved for paralogs, with some members of all four gene classes (*ClPIb*, *ClAGb*, *ClAGL11b*, *ClSEPc*, *ClSEPg*) showing a consistent expression pattern with lower expression levels and broader expression domains (**Figure 5**). In Asteraceae (**Figure 7**), expansions and duplications of ABC(D)E class genes are widespread. Expansions of A-, B-, C-, and E-class genes were also found in *G*. *hybrid*. Some members have low expression levels and broad expression domains. Inhibition of these members results in little phenotype alteration (Broholm et al., 2010; Ruokolainen et al., 2010a; Ruokolainen et al., 2010b; Zhang et al., 2017). These results suggest that, in response to the expansion and duplication of ABC(D)E class genes, this strategy of subfunctionalization is conserved in Asteraceae.

### 4.4 Correlation between the number of MIKC and the capitulum types in Asteraceae

The number of MIKCc gene subfamily members showed significant differences among Asteraceae plants with distinct capitulum types (**Figure 7B**). The largest account of MIKCc was 85 in *L. sativa* which has the ligulate type capitulum. The smallest number of MIKCc was in the discoid type capitulum species *C. cardunculus*, at 23. ANR1, SVP, SOC1 FLC, and SEP clades all underwent a significant expansion in *L. sativa* due to a specific whole genome triplication (WGT) event, with many MADS-box genes distributed within the triplication region (Reyes-Chin-Wo et al., 2017). In *H. annuus*, which also underwent WGT (Badouin et al., 2017), the number of MIKCcs was also significantly higher than that in other Asteraceae. These results suggest that genome duplication events are the main reason for the expansion of MIKCc gene family in Asteraceae.

In perennial Brassicaceae plants (e.g. *Arabis alpine*), *FLC* expression is repressed by low temperatures of winter and reactivated in spring, conferring a seasonal flowering pattern to the plant (Leijten et al., 2018). In this study, the amount of FLC branch members were found to be significantly different among seven Asteraceae tested. Many *FLCs* retained in Asteraceae species with ray florets, although they did not need to undergo a low-temperature period to complete the flowering transition. Whereas in two discoid type capitulum species, *C. cardunculus* and *M. micrantha*, the *FLC* was significantly contracted or completely lost (**Figure 7B**). Previous studies have shown that *C. lavandulifolium* flowering is strictly dependent on short-day induction rather than low temperature (Fu et al., 2014), but four *FLC*-like (**Figure 3**) are retained in the genome and these genes contain a specific motif 10 (**Figure 3B**, **Supplementary Figure 1**). Transcriptomic data on the capitulum development stage showed that *ClFLCa* highly expressed at the stage of the florets and the corolla primordium differentiation (**Figure 4A**), which is the same expression pattern with A- and E-class genes (**Figure 4**). It might play a novel role in the capitulum. *CiMFL* in *Cichorium intybus*, the ortholog of *AtFLC*, was found to fail to recover the early flowering phenotype of the *flc3* mutant of *A. thaliana*, but instead resulted in abnormal leaf organ morphology. However, the morphological changes in the floral organs were not observed due to a continuous reduction in the *CiMFL* expression level during flowering in transgenic lines (Locascio et al., 2009). This result also suggests that members of the *FLC* in Asteraceae have a novel function in controlling organ morphology. Accordingly, we hypothesize that the *FLC* may have novel functions in the development of capitulum in Asteraceae, especially in the development of ray florets.

## 5 Conclusions

In this study, a total of 40 ClMIKCc proteins were identified and classified into 12 branches. Transcriptomic data and qRT-PCR indicated that most *ClMIKCc* are specifically expressed in the capitulum, and *ClFLCa* may have novel functions in regulating the differentiation and development of ray floret. *C. lavandulifolium* has a conservative coping strategy for the redundant MIKCc gene, where members of the same branch have similar expression patterns, but only one member is highly expressed. Y2H and LCA results revealed the interactions of ClMIKCc in different floral organs of *C. lavandulifolium*. That will help to determine the tetrameric model in *C. lavandulifolium* floral organs. The results of phylogenetic analysis of MIKCc in Asteraceae indicated that there may be a correlation between the number of SEPs and the complexity of capitulum structure, as the SEP clade was significantly expanded in Asteraceae with radial capitulum. However, FLC was almost lost in Asteraceae with discoid capitulum, which, combined with the expression pattern of *ClFLCa* in *C. lavandulifolium*, further indicates the potential role of FLC in ray floret development in Asteraceae.

## Data availability statement

The datasets presented in this study can be found in online repositories. The names of the repository/repositories and accession number(s) can be found in the article/**Supplementary Material**.

## Author contributions

SD conceived and designed the study. JL performed most of the experiments and data analysis. QZ performed part of gene expression analysis. JL wrote the manuscript, and DK, YP, and XW edited it. All authors contributed to the article and approved the submitted version.
