# Supplementary material for: Genome-wide identification of the MIKCc-type MADS-box gene family in Chrysanthemum lavandulifolium reveals their roles in the capitulum development
Source: Front Plant Sci. 2023 Mar 23;14:1153490. doi: 10.3389/fpls.2023.1153490 (PMC10076714; doi:10.3389/fpls.2023.1153490)
Supplement: Supplementary file 1 [file DataSheet_1.pdf]

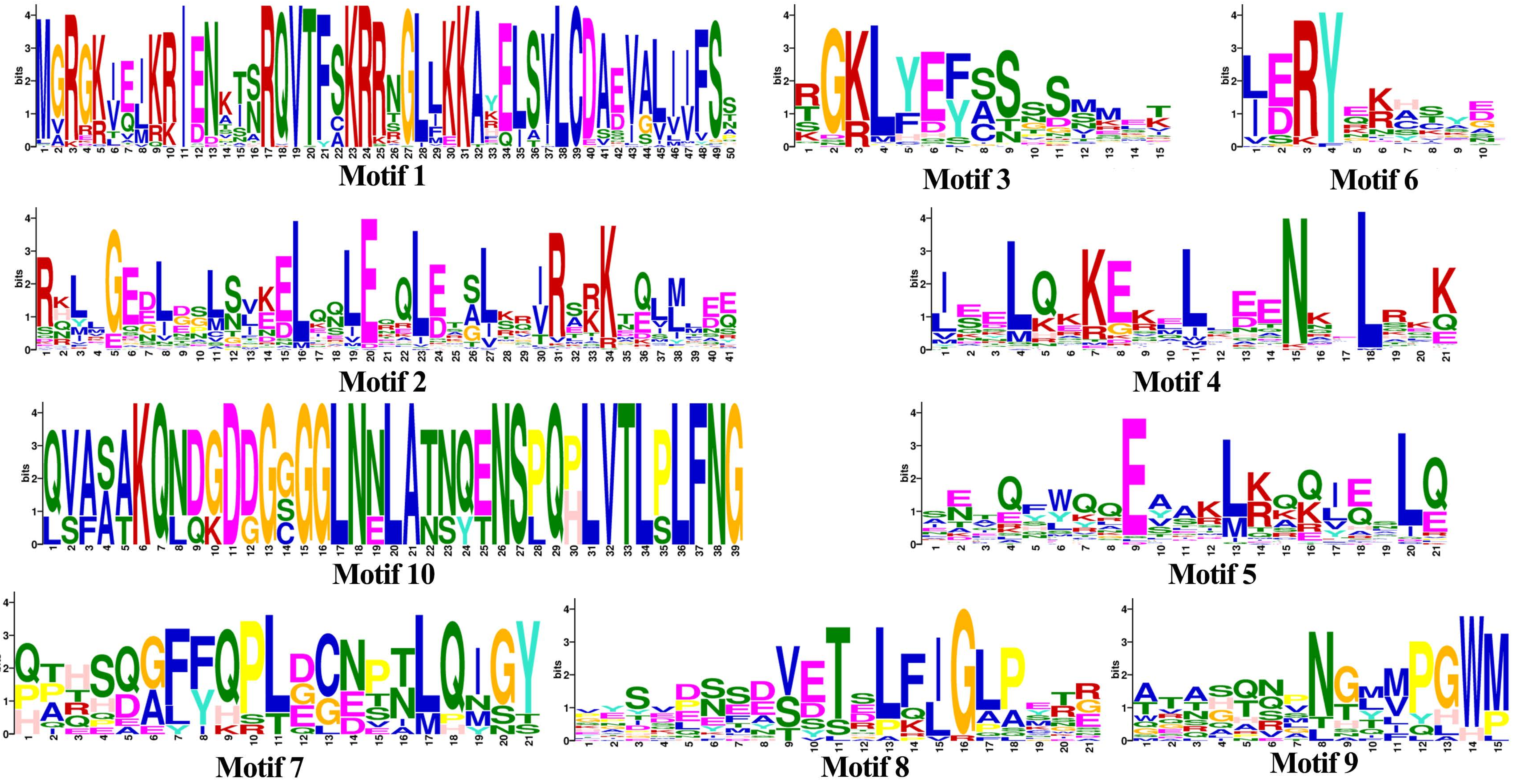

Supplementary Figure 1. Sequence logos of the *ClMIKCc* and *AtMIKCc* conserved motifs.

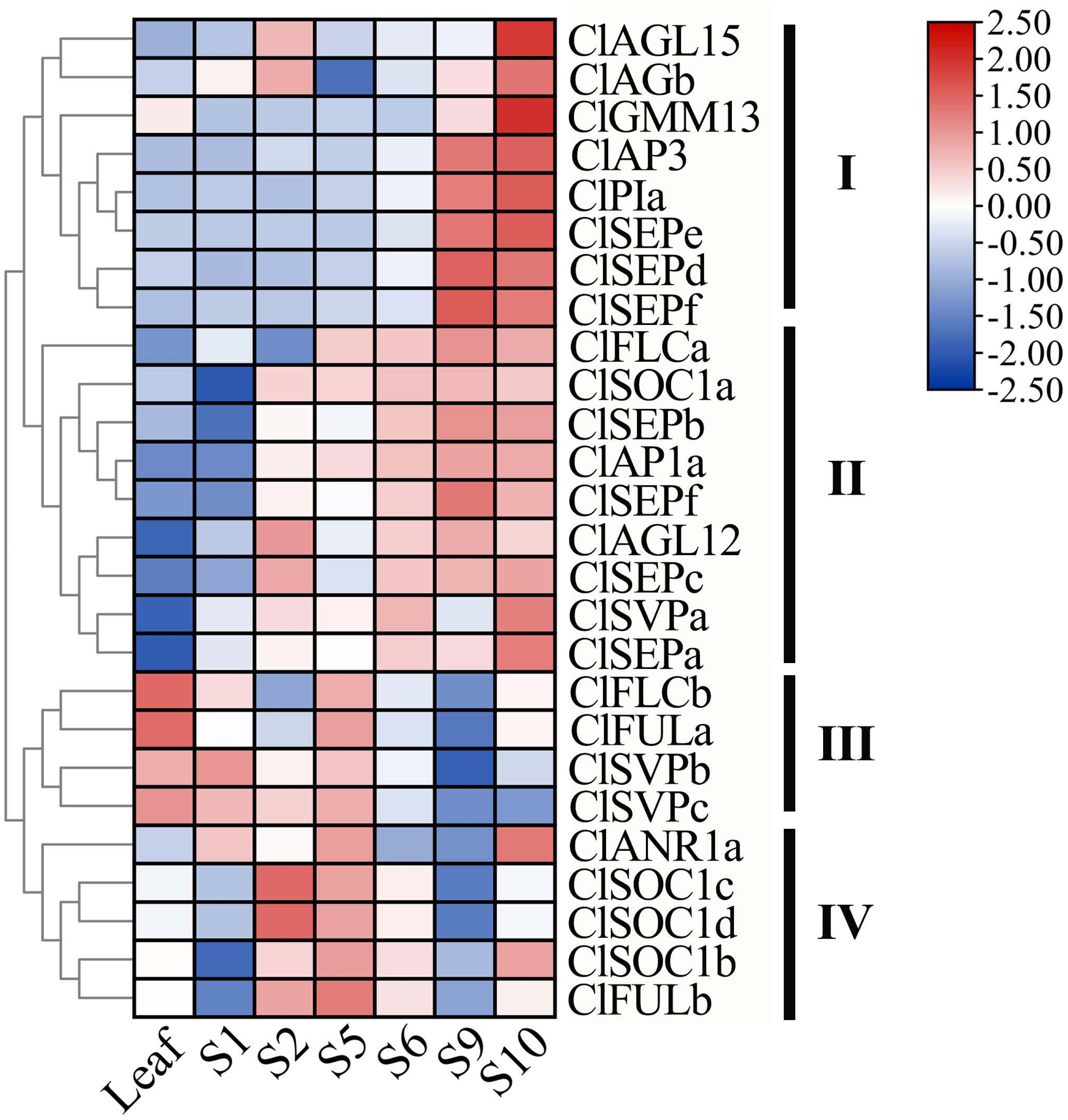

Supplementary Figure 2. Expression patterns of 26 *ClMIKCc* in six periods of capitulum development and leaves

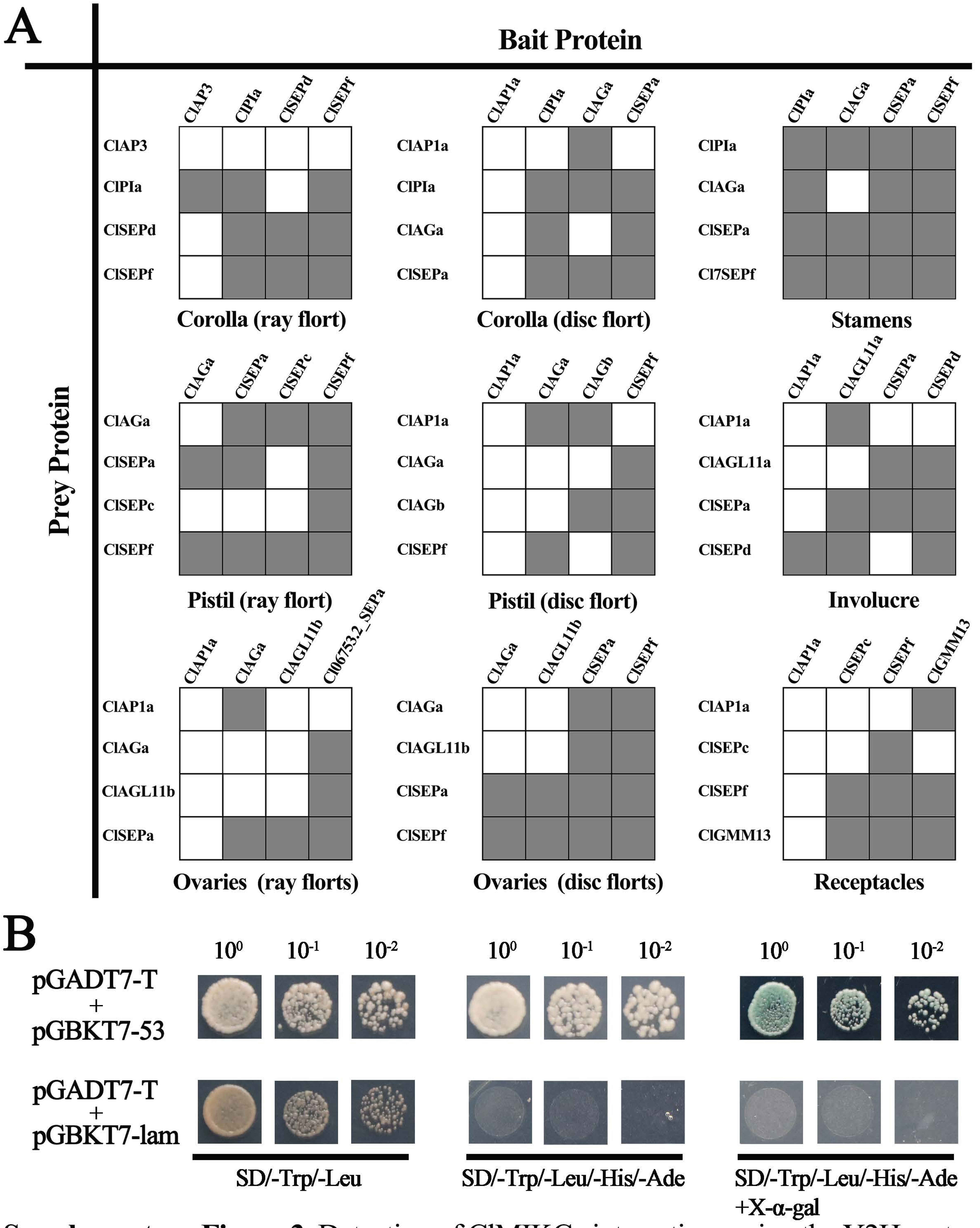

Supplementary Figure 3. Detection of ClMIKCc interactions using the Y2H system.

A. Pairwise interactions of ClMIKCc protein in nine floral organs.

The gray square indicates positive reciprocal interactions.

B. Positive (pGADT7+pGBKT7-53) and negative (pGADT7+GBKT7-lam) controls for Y2H assay.

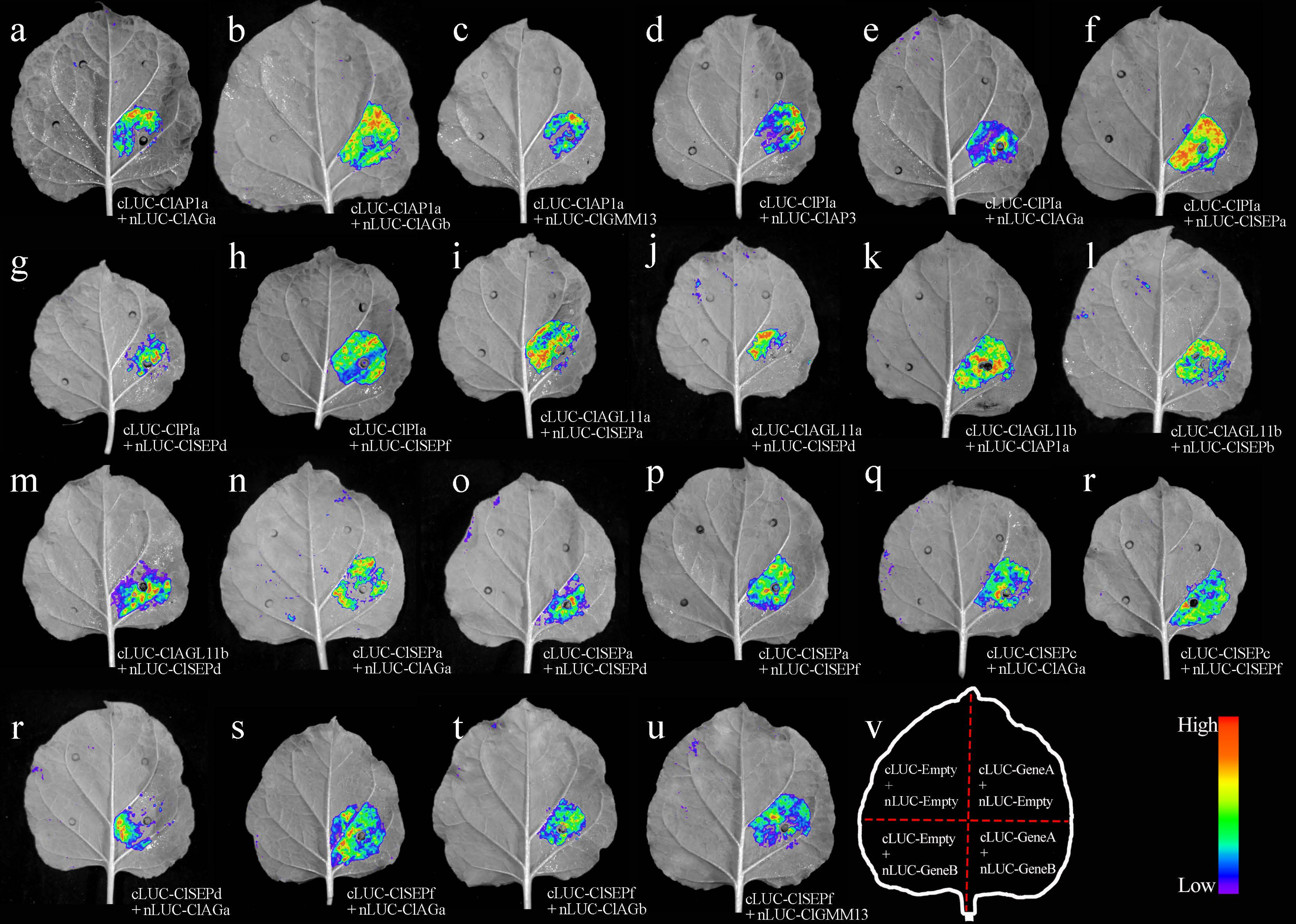

Supplementary Figure 4. Validation of ClMIKCc protein interactions using LCA in *N. benthamiana*.
